# Longitudinal changes in depressive symptoms associated with social isolation after the Great East Japan Earthquake in Iwate Prefecture: findings from the TMM CommCohort study

**DOI:** 10.1186/s12889-023-16082-z

**Published:** 2023-06-20

**Authors:** Yuka Kotozaki, Kozo Tanno, Kotaro Otsuka, Ryohei Sasaki, Makoto Sasaki

**Affiliations:** 1 Iwate Tohoku Medical Megabank Organization, Iwate Medical University, Iwate, Japan; 2 Department of Hygiene and Preventive Medicine, School of Medicine, Iwate Medical University, Iwate, Japan; 3 Department of Neuropsychiatry, School of Medicine, Iwate Medical University, Iwate, Japan; 4 Division of Physical Education, Department of Human Sciences, Iwate Medical University Center for Liberal Arts and Sciences, Iwate, Japan; 5 Division of Ultrahigh Field MRI, Institute for Biomedical Sciences, Iwate Medical University, Iwate, Japan

**Keywords:** Longitudinal change, Social isolation, Depressive symptoms, Past earthquake experiences.

## Abstract

**Background:**

Whether past disaster experiences affect the association between changes in social isolation and depressive symptoms is largely unknown. This study examined the association between changes in social isolation and depressive symptoms among survivors who experienced earthquake damage in the aftermath of the Great East Japan Earthquake (GEJE).

**Methods:**

We analyzed longitudinal data from 10,314 participants who responded to self-report questionnaires on the Lubben Social Network Scale-6 (LSNS-6) and the Center for Epidemiological Studies-Depressive Scale (CES-D) in both the baseline survey (FY2013 to FY2015) and follow-up survey (FY2017 to FY2019) after the GEJE. According to changes in the presence of social isolation (< 12 of LSNS-6) at two time points, participants were categorized into four groups: “not socially isolated,” “improved socially isolated,” “newly socially isolated,” and “continuously socially isolated.” At the follow-up survey, a CES-D score of ≥ 16 indicates the presence of depressive symptoms. The adjusted odds ratios (AORs) and 95% confidence intervals (CIs) were estimated using the logistic regression analysis to examine the influence of the change in social isolation over four years on depressive symptoms.

**Results:**

Participants who were newly socially isolated had a significantly higher prevalence of depressive symptoms than those who were not socially isolated (AOR = 1.89, 95% CI = 1.61 − 2.23). In addition, AORs were highest for those who were continuously socially isolated and had experienced house damage (AOR = 2.17, 95% CI = 1.73 − 2.72) and those who were newly socially isolated and had not experienced the death of family members due to the GEJE (AOR = 1.88, 95%CI = 1.60 − 2.22).

**Conclusion:**

Our longitudinal findings suggest that being newly or continuously socially isolated is associated with a risk of depressive symptoms, not only among those who had experienced house damage or the death of a family member, but also those who had not, in the disaster-affected area. Our study underlines the clinical importance of social isolation after a large-scale natural disaster and draws attention to the need for appropriate prevention measures.

**Supplementary Information:**

The online version contains supplementary material available at 10.1186/s12889-023-16082-z.

## Background

Natural disasters, such as large-scale earthquakes, can completely change the living environment and profoundly impact people’s minds and bodies [1, 2]. In the Great East Japan Earthquake (GEJE) of 2011, the massive earthquake and tsunami caused significant physical and personal damage, and those who were spared had to make drastic adjustments to their living conditions [3]. Moreover, due to the drastic changes in the living environment, social isolation occurred in the affected areas, and those who were socially isolated experienced deteriorating mental health [4–6].

Social isolation can negatively influence psychological health, leading to depressive symptoms [7, 8]. Oxman et al. reported that social isolation is among the most potent predictors of depressive symptoms among the elderly through data obtained from a probability sample of 2,806 individuals over the age of 65 living in the U.S. in Connecticut [9]. Santini et al. also found that social isolation predicted higher depressive symptoms in data from 3,005 adults aged 57–85 from the National Social Life, Health, and Aging Project (NSHAP) [10].

Only a few studies have examined longitudinal changes in social isolation after an earthquake [5, 11]. For instance, Sone et al. analyzed changes in social isolation between 2011 and 2014 using longitudinal data from 959 participants in a community-based survey in Miyagi Prefecture. They found that 11.1% of participants were “socially isolated,” 10.0% were “not socially isolated,” and 14.9% remained “socially isolated” [5]. In addition, among the participants who had psychological distress at the baseline, the rate of improvement of psychological distress was significantly higher in participants who “remained not socially isolated” [5]. Four to six years after the GEJE, Sekiguchi et al. used propensity score analysis to examine changes in social isolation based on whether or not people moved into publicly reconstructed housing. They reported a significant rise in the prevalence of social isolation among those who moved into newly constructed housing [11]. Meanwhile, longitudinal changes in depressive symptoms after the earthquake have been examined in several studies, but the findings are inconsistent [12–14]. For instance, Goenjian et al. reported that depression scale scores improved significantly over time based on data collected 1.5 and 4.5 years post-trauma from 29 individuals who had suffered mild earthquake trauma in the 1988 Spitak earthquake [12]. Kino et al. examined long-term trends in mental health disorders after the GEJE using data from 1,735 community residents of Iwanuma City, Miyagi Prefecture, and reported that the prevalence of depressive symptoms remained stable at approximately 29% in both 2013 and 2016 [13]. Meanwhile, Hikichi et al. reported that the mean scores of depressive symptoms increased slightly over time for 2.5 years and 5.5 years after the earthquake based on data from 2,664 community residents in Iwanuma City, Miyagi Prefecture, after the GEJE [14]. However, how disaster situations such as house damage and the death of family members affect the association between subsequent social isolation and depressive symptoms is largely unclear.

Thus, we aimed to examine the longitudinal change association between social isolation and depressive symptoms due to the experience of earthquake damage in the aftermath of the GEJE.

## Methods

### Study population

This study is part of the Tohoku Medical Megabank Project Community-Based Cohort Study (TMM CommCohort Study) [15, 16]. The baseline survey was conducted between 2013 and 2016 in Miyagi and Iwate Prefectures in Japan. This study employed data from individuals living in 20 municipalities, including 12 municipalities bordering the coast of Iwate Prefecture and eight surrounding areas in Iwate Prefecture. Study participants were recruited at municipal specific health check-up sites and at our facility. Recruitment was done by calling for participation in the cohort study at municipal specific health check-up sites in selected municipalities or using mass media such as newspapers and flyers to recruit participants [15, 16]. The inclusion criteria were persons aged 20 years or older who were registered in the basic resident register of all municipalities in Iwate Prefecture at the time of enrollment. This manuscript adheres to the STROBE guidelines (see Supplementary Table 1).

We followed 32,320 of the 32,919 participants in the baseline surveys, excluding six dual enrollees and 593 individuals who withdrew consent. Among these, 20,810 individuals participated in the second survey (FY2017 to FY2019). Of these participants, we excluded 210 participants who did not return the questionnaire, 1,500 with missing data on the Lubben Social Network Scale (LSNS-6), 971 with missing data on the Center for Epidemiologic Studies-Depression Scale (CES-D), and 1,424 with missing data in the covariate variables. We also referred to the study by Marx et al. [17] and excluded 6,381 participants who have co-morbid disease (anxiety disorders, bipolar disorder, schizophrenia, dementia, Parkinson’s disease, migraine, cerebrovascular disease, hypertension, diabetes, and hyperlipidemia), which was picked up in the medical history section of our questionnaire. Consequently, we analyzed 10,314 participants (3,144 men and 7,170 women; mean age at baseline survey 57.0 ± 11.8 years).

### Measurements

### Depressive symptoms in baseline and second survey

Depressive symptoms were assessed using the CES-D [18]. The reliability and validity of the Japanese version of the CES-D were confirmed [19]. Depressive symptoms were defined as a CES-D score of ≥ 16 [18, 19].

### Social isolation in baseline and second survey

Social isolation was assessed using the LSNS-6 [20, 21]. The LSNS-6 contains six items on social connections (three questions about family ties and three questions about friendship ties). On a 6-point Likert scale, each item is ranked. The LSNS-6 scores range from 0 to 30. The reliability and validity of the LSNS-6’s Japanese version have been confirmed [22]. A score of < 12 [21, 22] indicates social isolation.

### House damage and death of family members due to the GEJE in baseline survey

We used six options to assess house damage caused by the GEJE: (1) totally damaged (including all outflows), (2) seriously damaged, (3) half-damaged, (4) partially damaged, (5) no damage, and (6) non-residence. These options were further classified as damaged (totally damaged, seriously damaged, half-damaged, and partially damaged) or undamaged (no damage or non-residence). Participants responded affirmatively or negatively to questions regarding the death of family members due to the GEJE.

### Covariates

The following demographic characteristics were used as covariates in the analysis. These factors are linked to depressive symptoms and social isolation [23–25]: age (continuous), sex, education level (junior high school, high school, college or university or higher, and other), marital status (unmarried or married), number of household members (living alone or ≥ 2), work status (unemployed or employed), smoking habits (nonsmoker or current smoker), drinking habits (nondrinker or current drinker), body mass index (BMI, < 18.5, 18.5 to < 25, or ≥ 25 kg/m^2^), and insomnia. Insomnia was defined as a score of ≥ 6 on the Athens Insomnia Scale [26, 27]. The educational level was asked only in the baseline survey. Other variables used were the values answered during the second survey.

### Statistical analysis

First, McNemar’s test was used to compare the prevalence of depressive symptoms and social isolation in the baseline and second surveys. The participants were categorized into four groups based on their social isolation level (low: LSNS-6 < 12) in the baseline and second survey: (1) not socially isolated (low and low), (2) improved socially isolated (high and low), (3) newly socially isolated (low and high), and (4) continuously socially isolated (high and high). Second, we used the analysis of variance for continuous variables and the chi-square test for categorical variables to compare the characteristics of the four groups. Third, the association between social isolation and depressive symptoms was examined. We used logistic regression analysis to estimate the multivariate adjusted odds ratios (AORs) and 95% confidence intervals (CIs) after adjusting for covariates to indicate whether the change in social isolation was associated with depressive symptoms after four years. We also conducted stratified analyses by sex and age group (< 65 or ≥ 65 years) and by risks of the baseline depressive symptoms (higher or lower risk).

We hypothesized that the experience of damage from disasters, such as the house damage or the death of family members due to the GEJE, might be a potential risk for social isolation, which might influence depressive symptoms. To investigate how the disaster situation influences the relationship between social isolation and depressive symptoms, we also stratified house damage and the death of family members. In addition, to examine whether social isolation has different effects on depressive symptoms depending on the presence or absence of experiences such as house damage or the death of family members due to the GEJE, the main effect and interaction terms were simultaneously fed into the model. To examine the interaction between house damage (or death of family members due to the GEJE) and social isolation, the product term of house damage (or death of family members due to the GEJE) and social isolation was put into a model that was not stratified by house damage (or death of family members due to the GEJE). The results shows p values for the product term of house damage (or death of family members due to the GEJE) and social isolation.

Moreover, to avoid the possibility of reverse causalities, we performed the same analysis, excluding 2,730 participants who have depressive symptoms in the baseline survey.

All statistical analyses were conducted using SPSS version 25.0 for Windows (IBM, Tokyo, Japan). P values of < 0.05 indicated statistical significance.

## Results

Table 1 presents the distribution of the changes in depressive symptoms. The prevalence of depressive symptoms did not differ significantly between the baseline and second surveys (26.5% and 25.7%, p = 0.10). Regarding the change in the prevalence of depressive symptoms during the same period, 10.7% of the participants showed an improvement, 9.9% showed deterioration, and 15.8% remained high in depressive symptoms. Meanwhile, Table 2 presents the distribution of changes in the prevalence of social isolation. The prevalence of social isolation differed significantly between the baseline and second survey (27.6% and 31.0%, p < 0.001).

**Table 1:** Distribution of change in depressive symptoms

		Second surveys		*P* value
		Depressive Symptoms -	Depressive Symptoms +	
Baseline surveys	Depressive symptoms –	Remained low depressive symptoms n = 6,561 (63.6%)	Deterioration n = 1,023 (9.9%)	0.103
	Depressive symptoms +	Improvement n = 1,099 (10.7%)	Remained high depressive symptoms n = 1,631 (15.8%)	

Bonferroni correction, P < 0.05

**Table 2:** Distribution of change in social isolation

		Second surveys		*P* value
		No social isolation	Social isolation	
Baseline surveys	No social isolation	Not socially isolated n = 6,247 (60.6%)	Newly socially isolated n = 1,224 (11.9%)	＜0.001
	Social isolation	Improved socially isolated n = 865 (8.4%)	Continuously socially isolated n = 1,978 (19.2%)	

Bonferroni correction, P < 0.05

Table 3 displays baseline characteristics according to changes in social isolation. Compared to other participants, those who are continuously socially isolated were more likely to be younger, men, unmarried, living alone, exhibiting depressive symptoms, and suffering from insomnia. In addition, newly socially isolated participants were more likely to be highly educated, current smokers, current drinkers, and thin compared with other participants.

**Table 3:** Baseline characteristics according to change in social isolation (n = 10,314)

		Social isolation change				
		Not socially isolated	Improved socially isolated	Newly socially isolated	Continuously socially isolated	P value
No. of participants		6,267	865	1,224	1,978	
Age in baseline surveys		58.0 (11.9)	56.4 (12.0)	55.8 (11.1)	55.0 (11.8)	< 0.001
Sex (%)	Men	29.5	29.6	32.6	32.9	0.001
Education (%)	Junior high school	18.8	17.8	15.0	16.9	0.024
	High school	48.3	49.0	52.0	52.0	
	College / university or higher	31.8	31.7	32.1	29.9	
	Other	1.1	1.5	0.9	1.2	
Marital status (%)	Unmarried	19.4	23.0	23.4	31.1	< 0.001
Number of household members (%)	Living alone	7.1	8.1	7.1	9.8	0.001
Work status (%)	Unemployed	40.6	40.5	38.5	41.0	0.524
Smoking habits (%)	Current smoker	12.3	12.3	15.0	13.7	0.040
Drinking habits (%)	Current drinker	48.4	48.5	51.1	45.0	0.007
BMI (%)	< 18.5 kg/m^2^	6.2	6.9	8.3	8.1	0.015
	18.5 to < 25 kg/m^2^	70.5	70.9	68.8	70.4	
	≥ 25 kg/m^2^	23.3	22.2	22.9	21.4	
Depressive symptoms (%)	≥ 16	19.9	34.3	30.1	41.7	< 0.001
Insomnia (%)	≥ 6	18.3	28.0	23.7	31.1	< 0.001
House damage (%)		35.1	35.0	35.0	34.4	0.963
The death of family members due to the GEJE (%)		4.5	5.8	4.2	4.9	0.298

BMI, body mass index; GEJE, Great East Japan Earthquake

Responses for education, house damage, and death of family members due to the GEJE were obtained from the baseline survey

Bonferroni correction, P < 0.05

The AORs (95% CI) for depressive symptoms according to changes in social isolation are shown in Table 4. Participants who are newly and continuously socially isolated had a significantly higher prevalence of depressive symptoms than those who are not socially isolated. This tendency did not change in the stratified analysis by sex, age group, and risks of the baseline depressive symptoms (Supplementary Tables 2, 3, and 4).

**Table 4:** AOR and 95%CI of depressive symptoms according to change in social isolation

			Model 1	Model 2	Model 3	Model 4
		Cases / Participants	Crude OR (95%CI)	AOR (95%CI)	AOR (95% CI) [Model 2 + house damage]	AOR (95% CI) [Model 3 + death of family members due to the GEJE]
Not socially isolated		1,241 / 6,267	1.00 (Ref.)	1.00 (Ref.)	1.00 (Ref.)	1.00 (Ref.)
Improved socially isolated		297 / 865	1.60 (1.36–1.88)	1.15 (0.95–1.40)	1.15 (0.95–1.40)	1.15 (0.95–1.40)
Newly socially isolated		368 / 1,224	2.24 (1.96–2.56)	1.89 (1.61–2.22)	1.89 (1.61–2.23)	1.89 (1.61–2.23)
Continuously socially isolated		824 / 1,978	2.77 (2.48–3.09)	1.87 (1.63–2.14)	1.87 (1.63–2.14)	1.87 (1.63–2.14)
Covariates						
Age				0.99 (0.98–1.02)	0.99 (0.98–1.00)	0.99 (0.98–1.00)
Sex	Men			1.22 (1.06–1.40)	1.22 (1.06–1.39)	1.22 (1.06–1.39)
Education	High school			0.93 (0.80–1.09)	0.93 (0.80–1.09)	0.94 (0.80–1.09)
	College / university or higher			0.88 (0.74–1.04)	0.88 (0.74–1.05)	0.88 (0.74–1.05)
	Other			0.84 (0.48–1.44)	0.84 (0.48–1.44)	0.84 (0.48–1.44)
Marital status	Unmarried			1.31 (1.13–1.52)	1.31 (1.13–1.52)	1.31 (1.13–1.52)
Number of household members	Living alone			1.21 (0.98–1.48)	1.21 (0.98–1.48)	1.21 (0.98–1.48)
Work status	Unemployed			1.10 (0.98–1.25)	1.10 (0.98–1.25)	1.10 (0.98–1.25)
Smoking habits	Current smoker			1.01 (0.84–1.22)	1.01 (0.84–1.22)	1.01 (0.84–1.22)
Drinking habits	Current drinker			1.11 (0.99–1.24)	1.11 (0.99–1.24)	1.11 (0.98–1.24)
BMI	< 18.5 kg/m^2^			1.10 (0.89–1.37)	1.10 (0.89–1.37)	1.10 (0.89–1.37)
	≥ 25 kg/m^2^			1.07 (0.95–1.22)	1.08 (0.95–1.22)	1.08 (0.95–1.22)
Depressive symptoms in the baseline survey	≥ 16			6.52 (5.83–7.29)	6.52 (5.83–7.29)	6.52 (5.83–7.30)
Insomnia	≥ 6			6.05 (5.40–6.78)	6.05 (5.41–6.78)	6.05 (5.41–6.78)
House damage					0.99 (0.88–1.11)	0.99 (0.88–1.11)
The death of family members due to the GEJE						0.96 (0.74–1.24)

AOR, adjusted odds ratio; CI, confidence interval; Ref, reference

The AORs were adjusted for age, sex, education, marital status, number of household members, work status, smoking habits, drinking habits, BMI, insomnia, house damage, death of family members due to the GEJE, and depressive symptoms in the baseline survey

Bonferroni correction, P < 0.05

Table 5 shows that participants who are newly and continuously socially isolated who had experienced house damage had a significantly higher prevalence of depressive symptoms than those who not socially isolated [newly socially isolated: AOR (95% CI) = 1.92 (1.47–2.52); continuously socially isolated: AOR (95% CI) = 2.17 (1.73–2.72)]. In addition, participants who are newly socially isolated and those who continuously socially isolated who did not experienced house damage had a significantly higher prevalence of depressive symptoms than those who are not socially isolated [newly socially isolated: AOR (95% CI) = 1.88 (1.53–2.30); continuously social isolated: AOR (95% CI) = 1.73 (1.45–2.30)]. There was no interaction between house damage and social isolation (p = 0.285).

**Table 5:** AOR and 95% CI of depressive symptoms according to social isolation change by house damage

		Cases / Participants	AOR (95%CI)	*P* value	*P* for interaction
Undamaged	Not socially isolated	752 / 4,056	1.00 (Ref.)		0.285
	Improved socially isolated	149 / 562	1.19 (0.93–1.53)	0.161	
	Newly socially isolated	271 / 795	1.88 (1.53–2.30)	< 0.001	
	Continuously socially isolated	481 / 1,297	1.73 (1.45–2.30)	< 0.001	
Damaged	Not socially isolated	451 / 2,191	1.00 (Ref.)		
	Improved socially isolated	90 / 303	1.09 (0.79–1.50)	0.594	
	Newly socially isolated	155 / 429	1.92 (1.47–2.52)	< 0.001	
	Continuously socially isolated	305 / 681	2.17 (1.73–2.72)	< 0.001	

AOR, adjusted odds ratio; CI, confidence interval; Ref, reference

The AORs were adjusted for age, sex, education, marital status, number of household members, work status, smoking habits, drinking habits, BMI, insomnia, death of family members due to the GEJE, and depressive symptoms in the baseline survey

Bonferroni correction, P < 0.05

Table 6 demonstrates that participants who fall into the improved socially isolated, newly socially isolated, and continuously socially isolated who had not the death of family members due to GEJE had a significantly higher prevalence of depressive symptoms than those who no socially isolated [AOR (95% CI) = 1.23 (1.01 − 1.50), 1.88 (1.60 − 2.22), and 1.88 (1.63 − 2.16), respectively]. Participants who are improved socially isolated who had the death of family members due to the GEJE also had a significantly lower prevalence of depressive symptoms than those who are not socially isolated [AOR (95% CI) = 0.37 (0.14 − 0.97)]. There was no interaction between the death of family members due to the GEJE and social isolation (p = 0.079).

**Table 6:** AOR and 95%CI of depressive symptoms according to social isolation change by deaths of family members due to the GEJE.

		Cases / Participants	OR (95%CI)	*P* value	*P* for interaction
No death of family members due to the GEJE	Not socially isolated	1,139 / 5,969	1.00 (Ref.)		0.079
	Improved socially isolated	226 / 815	1.23 (1.01–1.50)	0.047	
	Newly socially isolated	402 / 1,172	1.88 (1.60–2.22)	< 0.001	
	Continuously socially isolated	750 / 1,882	1.88 (1.63–2.16)	< 0.001	
Death of family members due to the GEJE	Not socially isolated	64 / 278	1.00 (Ref.)		
	Improved socially isolated	13 / 50	0.37 (0.14–0.97)	0.044	
	Newly socially isolated	24 / 52	1.96 (0.89–4.35)	0.097	
	Continuously socially isolated	36 / 96	1.77 (0.91–3.41)	0.091	

GEJE, Great East Japan Earthquake; AOR, adjusted odds ratio; CI, confidence interval; Ref, reference

The AORs were adjusted for age, sex, education, marital status, number of household members, work status, smoking habits, drinking habits, BMI, insomnia, house damage, and depressive symptoms in the baseline survey

Bonferroni correction, P < 0.05

Moreover, we performed the same analysis, excluding 2,730 participants who had depressive symptoms in the baseline survey. Participants who are newly and continuously socially isolated had a significantly higher prevalence of depressive symptoms than those who are not socially isolated after adjusting for all the covariates (Supplementary Table 5). The AORs (95% CI) for depressive symptoms according to social isolation change stratified by sex are shown in Supplementary Table 6. There was no interaction between age group and social isolation (p = 0.322). The AORs (95% CI) for depressive symptoms according to social isolation change stratified by age group are shown in Supplementary Table 7. There was no interaction between age group and social isolation (p = 0.346). The AORs (95% CIs) for depressive symptoms according to house damage and social isolation change are shown in Supplementary Table 8. Participants who are newly and continuously socially isolated who had experienced house damage had a significantly higher prevalence of depressive symptoms than those who are not socially isolated [newly socially isolated: AOR (95% CI) = 2.02 (1.43–2.85); continuously socially isolated: AOR (95% CI) = 2.40 (1.74–3.30)]. Furthermore, participants who are new and continuously socially isolated who did not experienced house damage had a significantly higher prevalence of depressive symptoms than those who are not socially isolated [newly socially isolated: AOR (95% CI) = 1.99 (1.55–2.57); continuously social isolated: AOR (95% CI) = 1.75 (1.39–2.20)]. There was no interaction between house damage and social isolation (p = 0.328). The AORs (95% CIs) for depressive symptoms according to the presence or absence of death of family members caused by the GEJE and social isolation change are shown in Supplementary Table 9. Participants in the category for improved, newly, and continuously socially isolated who had not the death of family members due to GEJE had a significantly higher prevalence of depressive symptoms than those who are not socially isolated [AOR (95% CI) = 1.34 (1.01 − 1.76), 1.98 (1.61 − 2.45), and 1.93 (1.60 − 2.34), respectively]. There was no interaction between the death of family members due to the GEJE and social isolation (p = 0.580).

## Discussion

This study examined the longitudinal association between social isolation and depressive symptoms after the GEJE. Although the prevalence of depressive symptoms did not differ (26.5% and 25.7%, respectively), the prevalence of social isolation differed between the baseline and second surveys (27.6% and 31.0%, respectively). Our data for social isolation showed that 11.9% of participants changed from being not socially isolated to being socially isolated, and 19.2% remained socially isolated. Using the LSNS-6, Sone et al. examined changes in the prevalence of social isolation and found that 11.1% of participants changed from being not socially isolated to being socially isolated and 14.9% remained socially isolated [5]. In terms of trends, our findings were consistent with those of the previous report and may be applicable to the progression of social isolation among survivors of other natural disasters.

We showed that the prevalence of depressive symptoms was significantly higher in participants who are newly and continuously socially isolated than in participants who are not socially isolated. Certain social settings can foster social isolation [28], and depression can be caused by social isolation [29]. The participants in our study experienced the GEJE, and it is conceivable that the disaster altered their subsequent living conditions. We consider that changes in the living environment and income may have created or sustained conditions that increased the likelihood of people isolating themselves in the aftermath of the disaster, resulting in the manifestation of depressive symptoms.

We also showed that among those who had experienced house damage, participants who are newly and continuously socially isolated had a significantly higher prevalence of depressive symptoms. Within a few years, individuals whose homes were damaged had to change their living environments, moving from shelters to temporary housing and then to disaster recovery housing [30]. As a result of repeated changes in their living environments, they may have become isolated and unable to maintain connections with others. In addition, the same tendencies were observed even among those who did not experience house damage. Individuals who did not suffer house damage may have had their previous social networks changed, triggered by the fact that there was no damage. Although others around them were affected by the damage, they may feel sorry and guilt that they were not affected and began to distance themselves from their surroundings, isolating themselves. Thus, changes in environmental factors due to earthquakes may have affected changes in social networks [5]. As a result, we consider the possibility that some people may have developed mental health problems. Depressive symptoms can be triggered by life environmental changes [31, 32].

In our study, among those who has not death of family members due to the GEJE, participants who were improved, newly, and continuously socially isolated had a significantly higher prevalence of depressive symptoms. When we examined the damage to these people’s homes, we found that they had less damage than those who were never isolated. This may be because, while those around them were grieving the loss of their immediate family members in the earthquake, they felt remorse and guilt that they had not suffered the same fate [30]. Moreover, they distanced themselves from their surroundings and became or remained socially isolated, which may have caused mental health problems and depressive symptoms. Therefore, regarding those who were socially isolated and had not experienced house damage or the death of family members due to the disaster, we believe that even if a disaster does not directly damage houses or people, the experience of a large-scale natural disaster that threatens daily life may have a latent impact on social isolation, which may become apparent over time.

We also found that those who improved from social isolation and had not experienced the death of family members due to the GEJE had a significantly higher OR for depressive symptoms. Depending on the individual, it can take years to recover from depressive symptoms [33]. While their living conditions have improved as time has passed since the disaster, and the isolated situation has improved, it is possible that the depressive symptoms may still be lingering. Meanwhile, the OR of depressive symptoms was significantly lower in those who were in the category of improved socially isolated among those who had death of family members. We believe that post-traumatic growth may have occurred. Post-traumatic growth refers to the growth of the human mind in the wake of a traumatic event that is extremely painful and distressing [34]. Experiencing the death of a family member is difficult to accept even in normal times, but it can be said that sudden separation due to an accident or disaster can often be a distressing and painful experience for those left behind to accept the fact. Post-traumatic growth is the ability to recapture the meaning of life and connections with others, be open to new possibilities, and grow as a person [34]. Even those who have been isolated for a time after the earthquake, or who have not been able to connect with others, can overcome their sadness by reevaluating the meaning of life and their relationships with others as time passes. As a result, we believe that both the state of isolation and the depressive symptoms improved.

The present study has some limitations. First, participants in this study may have had greater health awareness and better health status than the target population because they had voluntarily participated in health surveys. Therefore, the prevalence of depressive symptoms may have been underestimated, and the generalization of results must be considered carefully. Second, causality reversal, such as isolation resulting from the inability to leave the house due to illness, may exist. Finally, because we lack data prior to the GEJE, it is impossible to determine the extent to which social isolation levels prior to the GEJE impacted disaster response preparedness, which would impact depressive symptoms. Nevertheless, because our results depict a significant association, we believe that they are robust. This study is significant because few known studies have reported an association between social isolation changes and depressive symptoms after an earthquake using a population-based cohort study design and a large sample size.

## Conclusion

Our longitudinal findings suggest that newly or remained social isolation is associated with the risk of depressive symptoms only among those who had experienced house damage or death of family member but also among those who had not experienced house damage or death of family member among people who lived in the disaster-affected area. It is important to understand the long-term health status of residents who have experienced a life-threatening event such as a large-scale natural disaster, even if they did not suffer damage, such as house damage or the death of a family member.

## Electronic supplementary material

Below is the link to the electronic supplementary material.

Supplementary Material 1

## Data Availability

The TMM cohort data are available for a fee to researchers in Japan who have been approved by the prescribed registration and review procedures. For more information, visit: http://www.dist.megabank.tohoku.ac.jp/.

## Abbreviations

BMI: Body Mass Index
CES-D: The Center for Epidemiologic Studies Depression Scale
CI: confidence interval
GEJE: Great East Japan Earthquake
LSNS-6: Lubben Social Network Scale 6
OR: Odds ratio
SD: standard deviation
SPSS: Statistical package for social science
TMM CommCohort Study: Tohoku Medical Megabank Project Community-Based Cohort Study
